# Supplementary material for: Promising Therapeutic Efficacy of GC1118, an Anti-EGFR Antibody, against KRAS Mutation-Driven Colorectal Cancer Patient-Derived Xenografts
Source: Int J Mol Sci. 2019 Nov 24;20(23):5894. doi: 10.3390/ijms20235894 (PMC6928876; doi:10.3390/ijms20235894)
Supplement: Supplementary file 1 [file ijms-20-05894-s001.pdf]

Supplementary Materials

Supplemental Table 1. Expression of EGFR ligands in our colorectal cancer patient-derived xenograft panel

PDX ID	EGF (nM)	HB-EGF (nM)	TGF-α (nM)	BTC (nM)	AREG (nM)	EREG (nM)
**CRC-001T**	0	9.6	46.9	271.3	41.7	0
**CRC-002T**	0	4.4	42.7	190.8	25.3	28.6
**CRC-003T**	0.2	7.8	41	328.1	111.5	0
**CRC-004T**	0	0	10.6	95.8	15.93	26
**CRC-005T**	0	5.4	71.1	225.7	123	0
**CRC-006T**	0	12.3	56.9	270.7	157.2	1.5
**CRC-007T**	5.7	4.8	56.9	280.1	151.7	12.9
**CRC-008T**	0.6	13.1	79.1	323.2	203.8	3.5
**CRC-009T**	0	1.4	20.6	177.9	65.6	38.1
**CRC-010T**	4.9	11.1	61	297.4	203.1	0.3
**CRC-011T**	5.5	16.1	45.4	322.4	223.8	0
**CRC-012T**	7.2	17.3	51	335.1	236.5	0
**CRC-013T**	7.6	7.3	52	275.5	197.4	0
**CRC-014T**	0	7.5	44.6	251.2	177	0
**CRC-015T**	2.8	2	36.8	181.7	87.5	48.1
**CRC-016T**	0	9.2	90.6	252.7	81.3	0
**CRC-017T**	7.2	12.7	45.2	286.4	82.8	0.2
**CRC-018T**	1.8	11.5	59.4	265.8	232.9	0
**CRC-019T**	7	14.9	74	313.6	123.9	5.2
**CRC-020T**	10.2	3.6	31.8	216.8	143.4	0
**CRC-021T**	0	3.4	15.7	210.7	93.9	32.1
**CRC-022T**	0.1	8.9	49	266	193.4	0
**CRC-023T**	0	7.1	58	200.7	175.2	53
**CRC-024T**	0	5.7	59.3	285.5	44	0
**CRC-025T**	0	11.4	72.8	303.7	50.7	0
**CRC-026T**	3.4	14.4	80.2	319.4	78.4	0
**CRC-027T**	0	3.5	28	161.2	44.2	26.5
**CRC-028T**	5.4	16.3	48.2	355.2	318.2	2.2
**CRC-029T**	0	12	56.4	270.4	140.4	0
**CRC-030T**	5.2	8.7	46.3	251	136	0
**CRC-031T**	3.2	0	10.7	156.9	43.7	32.3
**CRC-032T**	11.5	8.9	47.2	267	149.3	0
**CRC-033T**	0.4	1.8	49.4	152.8	58.1	47.2
**CRC-034T**	0	0	8.3	186.3	90.3	32.8

**CRC-035T**	2.7	8.4	36.8	203.4	168.1	2.6
**CRC-038T**	0	9.2	81.7	270.9	255	0
**CRC-039T**	11	20.2	53.5	395.1	331.3	8.9
**CRC-040T**	0.2	9.3	69.3	367.4	324.8	14
**CRC-042T**	5.8	8.8	53.2	272.3	125	0
**CRC-043T**	2.1	15.3	41.5	282.8	264	0
**CRC-044T**	0.2	8.8	38.1	81.7	90.7	12.7
**CRC-045T**	13.4	2.1	41.1	190.2	220	0
**CRC-046T**	0	2.1	16.4	122	52.6	60.5
**CRC-055T**	0	2.4	32.8	188.7	164.3	17.2
**CRC-070T**	0	10.6	36.1	260.7	249.4	0
**CRC-071T**	4.8	12.7	52.4	328.6	149.3	10.6
**CRC-074T**	18.6	10	63.8	291.7	324.7	8.3
**CRC-077T**	0	10.7	58.6	268.1	317	0
**CRC-088T**	1.2	6	67	330.5	155.5	0
**CRC-091T**	8.1	1.4	21.8	137	135	28.2
**CRC-093T**	0	4	25.6	168.8	290.9	55

Supplemental Table 2. Colorectal cancer patient-derived xenografts used in *in vivo* efficacy test of Cetuximab and GC1118

	CRC-001T	CRC-003T	CRC-077T	CRC-026T	CRC-034T	CRC-088T	CRC-024T
KRAS status	Wild-type	Wild-type	Wild-type	G12D	G12V	G12V	G12D
Ratio of high-affinity to low-affinity ligands	7.86	3.35	1.08	5.25	1.56	2.57	7.97
Number of mice / group	5	5	7	5	5	4	4
Days of treatment	38	28	45	41	46	48	52
Tumor growth inhibition index of GC1118 (%, 1 mg/mouse)	36.7	-36.8	6.8	47.9	34.5	10.8	65.6
Tumor growth inhibition index of cetuximab (%, 1 mg/mouse)	72.4	-29.4	10.4	97.5	103.6	47.6	109.4

Supplemental Table 3. Statistical significance associated with Figure 3C

		Vehicle vs GC1118	Vehicle vs Cetuximab	GC1118 vs Cetuximab
CRC-001T	pAKT	1.45E-06	1.13E-05	0.221816
	pERK1/2	6.58E-06	3.08E-07	1.08E-07
CRC-003T	pAKT	0.000283	1.3E-11	2.47E-10
	pERK1/2	5.07E-13	2.23E-13	2.01E-07
CRC-077T	pAKT	0.575343	5.32E-06	1.75E-06
	pERK1/2	7.09E-16	2.23E-13	1.03E-10
CRC-026T	pAKT	6.86E-08	1.33E-06	0.044577
	pERK1/2	6.39E-17	1.36E-13	1.56E-09
CRC-034T	pAKT	0.000291	8.84E-07	0.000233
	pERK1/2	7.27E-12	6.28E-11	1.19E-08
CRC-088T	pAKT	0.478993	1.12E-11	2.45E-11
	pERK1/2	7.55E-12	8.63E-11	5.14E-08

Supplemental Table 4. Statistical significance associated with Figure 5B

pAKT				
	Cetuximab	BEZ235	GC1118	BEZ235 + GC1118
Vehicle	3.18E-16	1.42E-21	8.85E-20	1.00E-17
Cetuximab		3.591E-17	3.02E-15	1.65E-15
BEZ235			1.43E-06	5.69E-10
GC1118				3.69E-14
pERK1/2				
	Cetuximab	BEZ235	GC1118	BEZ235 + GC1118
Vehicle	1.80E-10	5.25E-12	4.80E-12	1.16E-12
Cetuximab		1.44E-07	2.47E-08	2.10E-14
BEZ235			0.33	2.77E-13
GC1118				2.23E-13

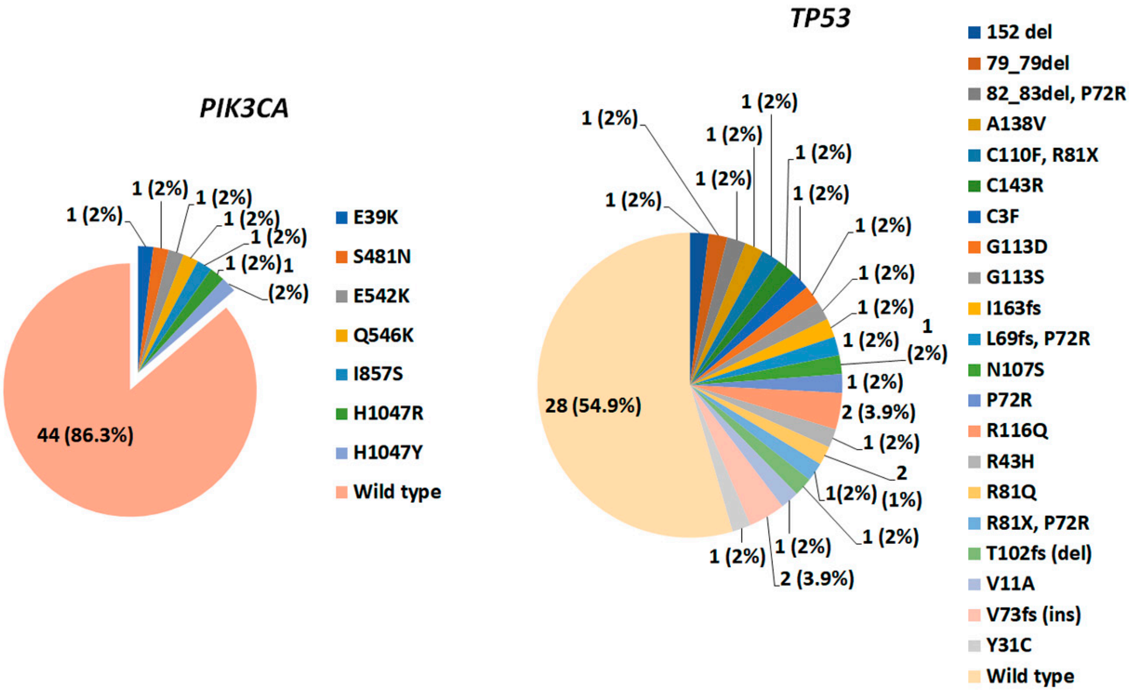

Supplemental Figure 1. Prevalence of phosphatidylinositol-4,5-bisphosphate 3-kinase catalytic subunit alpha (*PIK3CA*) (left panel) and tumor protein P53 (*TP53*) mutations (right panel) in our colorectal cancer patient-derived xenograft panel.

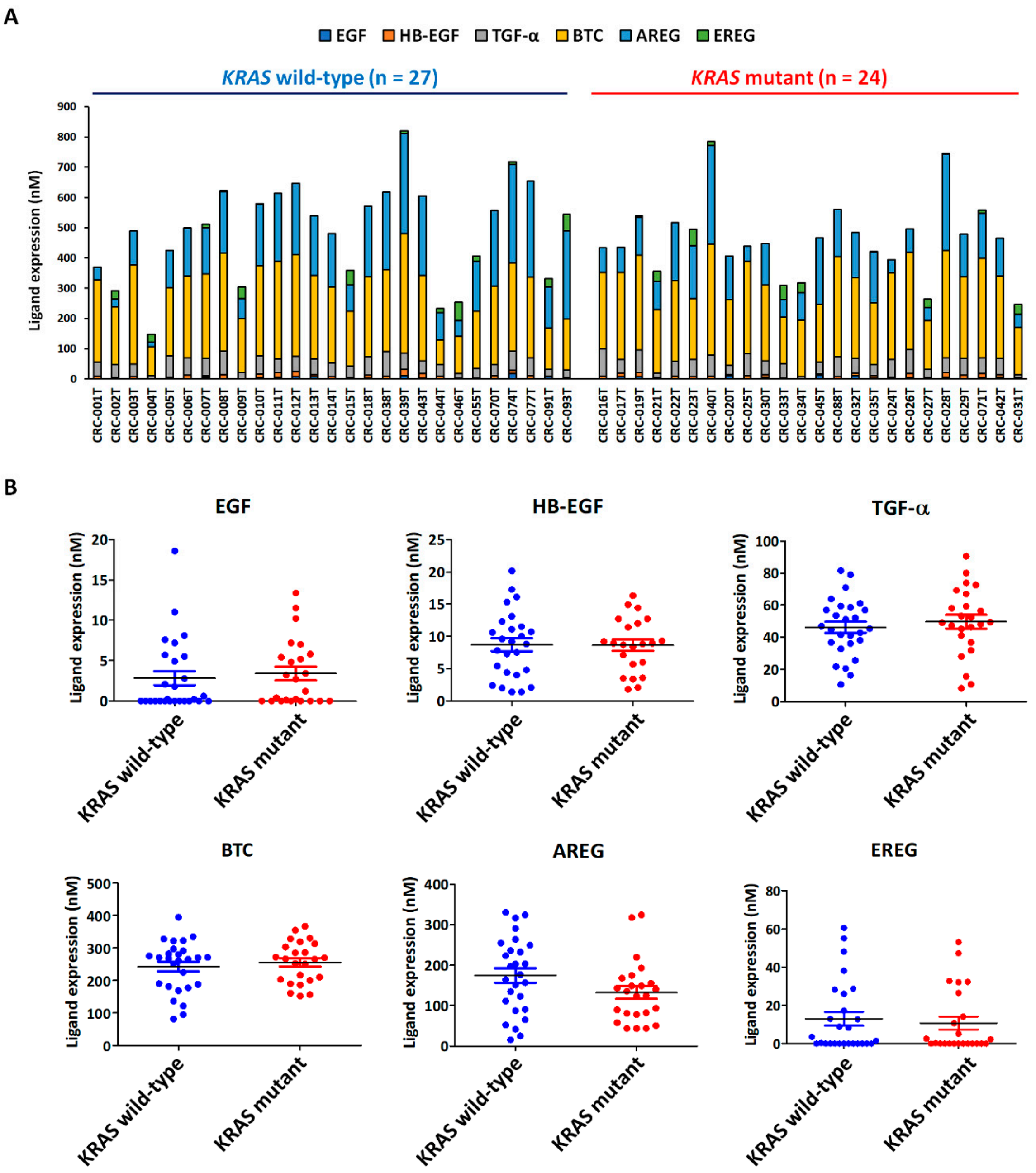

Supplemental Figure 2. Modulation of ligand expression from 51 our colorectal cancer (CRC) patient-derived xenografts (PDXs). **(a)** Enzyme-linked immunosorbent assay **(**ELISA) measurements of total ligand expression levels in 51 individual CRC PDXs. **(b)** Comparison of the expression of high- and low-affinity ligands for EGFR measured by ELISA according to their KRAS status in 51 individual CRC PDXs.

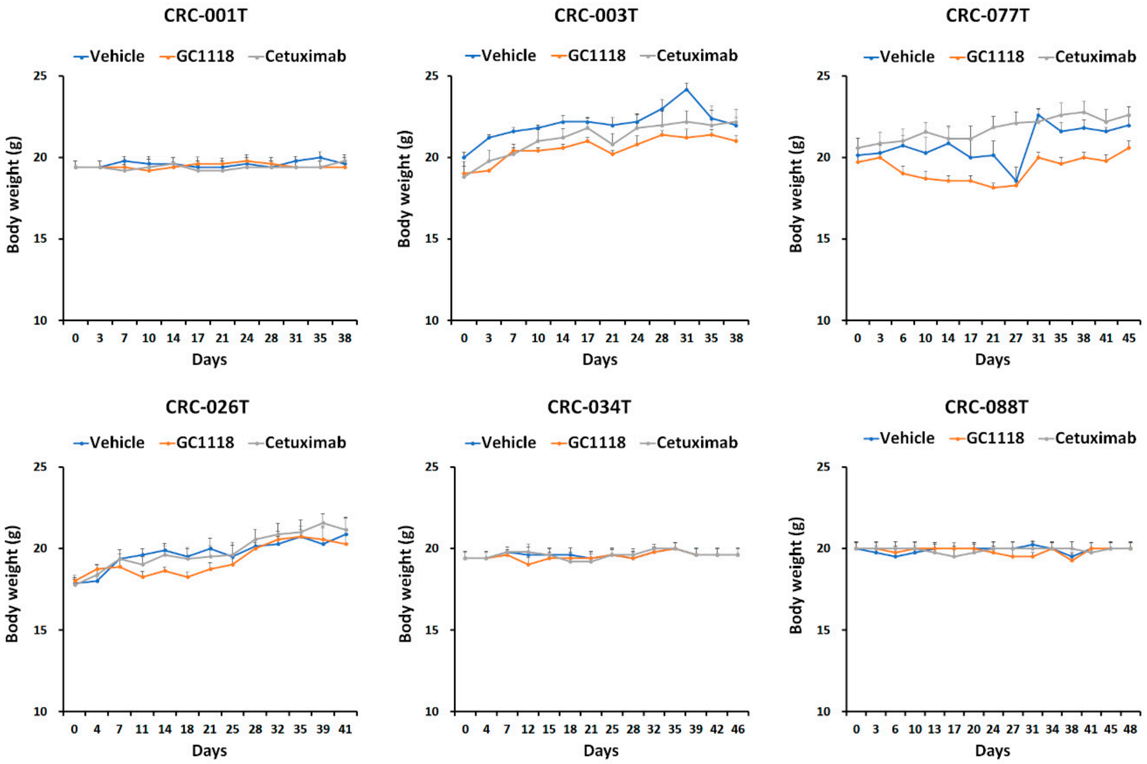

Supplemental Figure 3. Body weights of six colorectal cancer (CRC) patient-derived xenografts (PDXs) treated with vehicle, GC1118 and cetuximab. The results in the graph are shown as mean and SEM.

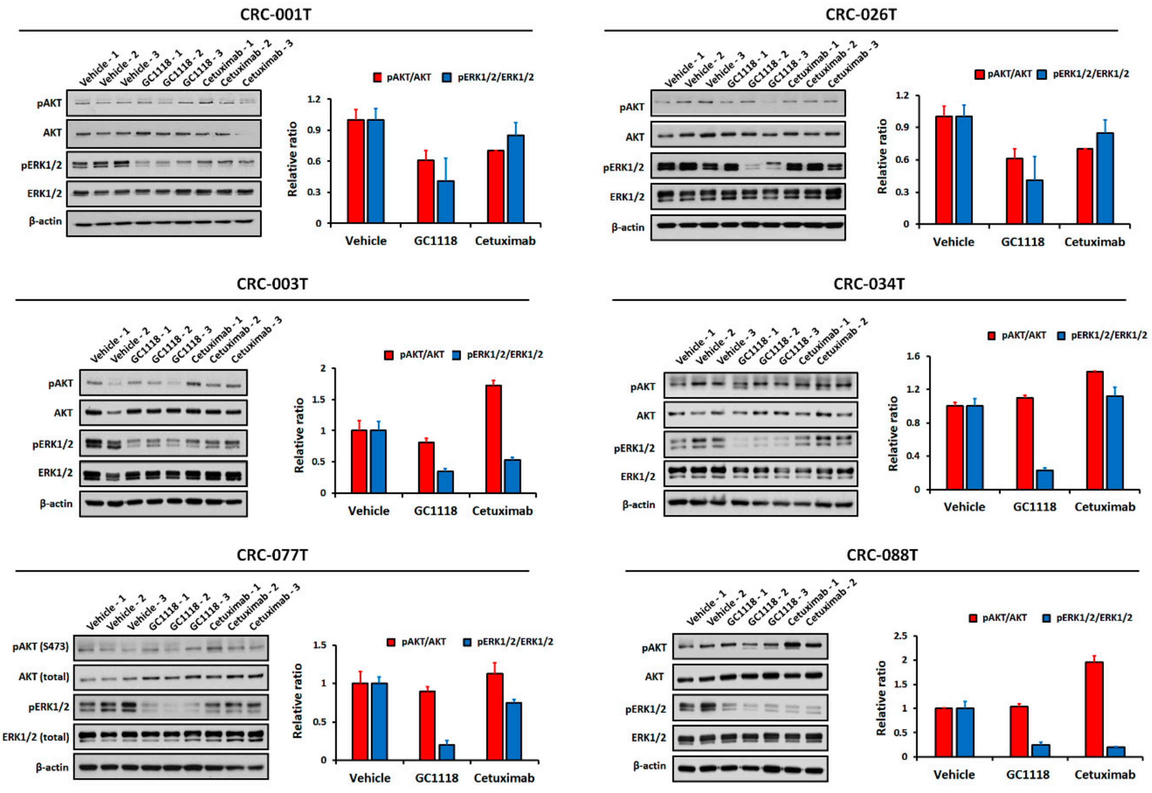

Supplemental Figure 4. The effects of vehicle, GC1118 and Cetuximab on six colorectal cancer (CRC) patient-derived xenografts (PDXs) evaluated by immunoblotting of tumor lysates prepared from harvested PDXs and the end of *in vivo* study. Analysis of signaling pathways by immunoblotting for AKT and ERK1/2 signaling activities in the CRC-024T PDX model treated with GC1118 and BEZ-235. For quantification, images were acquired and signal intensity of each protein band was quantified using the ImageJ software and normalized to β-actin. The activities of EGFR, AKT, and ERK1/2 were determined by normalization with their total pairs, namely p-AKT/AKT, and p-ERK1/2/ERK1/2, respectively. The results in the graph are shown as SEM.
